# Editorial: Pharmacology of new psychoactive substances

**DOI:** 10.3389/fphar.2023.1208957

**Published:** 2023-05-09

**Authors:** K. Lutfy, R. N. Pechnick, N. A. Darmani

**Affiliations:** ^1^ Department of Biotechnology and Pharmaceutical Sciences, College of Pharmacy, Western University of Health Sciences, Pomona, CA, United States; ^2^ Department of Basic Medical Sciences, College of Osteopathic Medicine, Western University of Health Sciences, Pomona, CA, United States

**Keywords:** psychoactive substances, synthetic psychoactive cathinones, THC-tetrahydrocannabinol, toxicology, central and peripheral action

Several classes of novel psychoactive substances have been introduced in illicit markets throughout the world and more are being developed. They present major law enforcement challenges and global public health threats. However, there is limited information on their harmful effects on the central nervous, cardiovascular, respiratory, and gastrointestinal systems. In particular, some phenethylamine derivatives are very potent psychedelic substances and may exert toxic effects at very low doses. Vorovbyeva and Kozlova reviewed three natural psychedelic drugs, psilocybin, ibogaine and N, N-dimethyltryptamine (DMT), in terms of their pharmacological properties and their therapeutic potential. Psilocybin primarily produces its psychedelic effects by acting as a partial agonist at serotonin 2A (5-HT2Ar), whereas DMT acts at a number of serotonin receptor subtypes. Ibogaine has a complicated pharmacology, interacting with a number of different neurotransmitter systems, including N-methyl-D-aspartate receptors, kappa- and mu-opioid receptors, sigma-2 receptors and nicotinic cholinergic receptors. Both psilocybin and DMT are relatively safe in humans, but ibogaine can decrease heart rate and cause life-threatening cardiac arrhythmias. All three drugs have shown some efficacy in the treatment of anxiety, depression and substance use disorders, but further evidence of safety and efficacy should be obtained. Syrová et al. report the pharmacokinetics and acute behavioural effects of 2C-B-Fly-NBOMe, an analog of popular psychedelic entactogen 2C-B (4-Bromo-2,5-dimethoxyphenethylamine), in male adult Wistar rats. Pharmacokinetic data revealed a serum peak concentration at 30 and a brain peak level at 60 min. The compound suppressed locomotor activity and, at a higher dose (5 mg/kg) caused prepulse inhibition, suggesting that it attenuates sensorimotor gating. It is well-known that some 4-substituted analogs of 1-(2,5-dimethoxyphenyl) isopropylamine (2,5-DMA) are serotonergic psychedelic agents that behave as agonists of the human serotonin 5-HT_2A_ (h5-HT2A) receptor and produce side-effects such as cardiac vulvulopathy via activation of h5-HT2B receptor. Hemanth et al. demonstrate rat and human 5-HT2A receptor binding data can be utilized for the estimation of affinity, but not the functional activity of phenylisopropylamines for h5-HT2B receptors. In fact, the authors demonstrate that both h5- HT2A and h5-HT_2B_ receptor affinities for 13 tested analogs of 2,5-DMA parallel their rat 5-HT_2A_ and 5-HT2B receptor affinities. The rat 5-HT_2_ receptor affinity was also related with the increased lipophilicity and electron withdrawing nature of the 4- position substituents of 2,5-DMA. Likewise, the latter properties correlate with their interactions at h5-HT2A and h5-HT2B receptors. In addition, they further confirmed that the 5-HT2B agonist action of these hallucinogens cannot be predicted on the basis of receptor affinity or the QSAR studies.

Synthetic cannabinoids (SCs) are new psychoactive drugs functionally similar to delta-9-tetrahydrocannabinol (THC) but with a higher binding affinity for cannabinoid CB1 and CB2 receptors and are currently used recreationally with potential health risks. JWH-122 and JWH-210 are naphthoylindole SCs and potent CB1 and CB2 receptor agonists. Clinical effects of SCs are mainly available from intoxication cases and surveys, with few human studies following controlled administration or observational studies using standardized measures of cardiovascular and subjective effects. Martinez et al. evaluated the acute pharmacological effects of inhaled JWH-122 (1 mg) and JWH-210 (2.25 mg) recreational consumption in a 4 h observational study and assessed their disposition in the oral fluid. JWH-122 but not JWH-210 caused significant increases in systolic and diastolic blood pressure, and heart rate. Moreover, only JWH-210 produced significant changes in subjective drug effects, similar to those induced by THC (intensity, high, and hunger). The maximal pharmacological effects and oral fluid concentrations mostly occurred 20 min after intake. The attained results demonstrate a different pattern of effects of these two SCs. In another report, Mendiguren et al. investigate the effect of cannabidiol (CBD) on the neuronal activity of dorsal raphe nucleus (DRN) 5-HT cells and its interaction with somatodendritic 5- HT1A autoreceptors. CBD is the main non-psychoactive cannabinoid found in the cannabis plant, evokes several pharmacological effects via the 5-HT1A receptor. The DRN is the principal serotonergic cluster in the midbrain that expresses the 5-HT1A receptor. These authors determined the effect of CBD on the firing activity of DRN 5-HT cells and the 5-HT1A autoreceptor activation by electrophysiological and calcium imaging techniques in rat brain slices. CBD did not change the firing rate of DRN 5-HT cells or the inhibitory effect of 5-HT. However, CBD reduced the inhibitory effects of selective 5-HT1A receptor agonists (8-OH-DPAT and ipsapirone) by over 50%. Although CBD failed to activate 5-HT1A autoreceptors, it hampered the inhibitory effect of selective 5-HT1A receptor agonists on the firing activity of DRN 5-HT cells through a mechanism that does not involve CB1, serotonergic 5- HT2A, or GABAA receptors. Their data suggest a negative allosteric modulation of DRN somatodendritic 5-HT1A receptor by CBD.

Cathinones are another class of new psychoactive agents. Poyatos et al. studied the effect of methylone, the most common synthetic cathinone, and MDMA in humans with a history of psychostimulant use. The subjects reported euphoria, increased heart rate and blood pressure, and wellbeing following both compounds. However, methylone worked faster but with a shorter duration of action, suggesting that methylone has the same pharmacological profile and possibly carries the same abuse potential as the parent compound. Zul Aznal et al. evaluated the effects of repeated administration (for 15 days) of mitragynine, the main alkaloid of kratom, and lyophilized kratom decoction on cognitive behaviours and brain metabolite profiles in adolescent (PND31) male Sprague Dawley rats. The authors showed that neither substance affects memory recognition in the novel object test. However, both substances caused a deficit in social interaction. Mitragynine also attenuated reference memory in the water maze test, which may be related to metabolite changes in the brain and involve different pathways.

These studies reveal the importance of further studying the effect of these compounds to expand our understanding of the pharmacology and toxicology of these drugs and to provide useful information regarding their deleterious effects to both clinicians and the governmental policymakers.

